# Depression, Anxiety and Stress Scales (DASS-21): Construct Validity Problem in Hispanics

**DOI:** 10.3390/ejihpe10010028

**Published:** 2020-01-08

**Authors:** Juan Aníbal González-Rivera, Orlando M. Pagán-Torres, Emily M. Pérez-Torres

**Affiliations:** School of Behavioral and Brain Sciences, Ponce Health Sciences University, 388 Zona Industrial Reparada 2, Ponce, PR 00716, USA; orlando.m.pagan.torres@gmail.com (O.M.P.-T.); emilyperez@psm.edu (E.M.P.-T.)

**Keywords:** anxiety, DASS-21, depression, stress, psychometric properties, validity

## Abstract

The main purpose of this research was to examine the construct validity of the Depression, Anxiety and Stress Scales (DASS-21) in order to determine whether it is able to adequately discriminate between symptoms of depression and anxiety in the Hispanic population in Puerto Rico. This study has an instrumental design. A total of 1073 Hispanics participated in this psychometric study. The results showed that the DASS-21 has serious psychometric deficiencies, especially related to the construct validity, as well as convergent and discriminatory validity. In addition, it was shown that DASS-21 do not replicate the three-dimensional structure of the original instrument in the Hispanic community. Finally, it was confirmed that the DASS-21 have difficulty in properly identifying and discriminating between symptoms associated with depression and anxiety in a Hispanic population.

## 1. Introduction

Mood disorders have the highest prevalence worldwide when compared to all other mental disorders, followed by anxiety disorders [1,2,3]. The scientific literature has thoroughly documented the comorbidity between anxiety and depression disorders in a variety of studies [4,5,6]. In fact, between 40% and 70% of people diagnosed with depression have been shown to simultaneously meet the diagnostic criteria for an anxiety disorder [7]. Similar studies have empirically shown the significant association between anxiety and depression, which is why it is often difficult to identify, treat, and distinguish both diagnoses [8].

For this reason, it is essential that at the beginning of any treatment, mental health professionals evaluate the presence and severity of symptoms associated with depression and anxiety in their patients [6]. Valid and reliable clinical measurement instruments are required to facilitate the diagnosis and treatment of people with simultaneous symptoms. This is only possible with instruments that properly discriminate between symptoms of anxiety and depression. In the case of Puerto Rico, very few researchers have conducted studies to analyze the psychometric properties of the most commonly used depression and anxiety measures in clinical scenarios [9,10,11]. Therefore, we do not find instrumental studies that evaluate the psychometric properties of the Depression, Anxiety and Stress Scales (DASS-21) in Puerto Rico, which is an instrument that in recent years has taken on become much more widely used for Spanish-speaking populations [12,13,14,15].

Given this lack of studies and with the certainty that DASS-21 are being used in Puerto Rico [16,17,18], several questions arise: Will the DASS-21 have adequate psychometric properties for the Puerto Rican population? Will they have the ability to adequately discriminate between clinical symptoms associated with depression and anxiety? To answer these questions, our study will analyze the construct validity of the Hispanic version of DASS-21 in a Puerto Rican adult sample.

### 1.1. Prevalence of Anxiety and Depression in Puerto Rico

The Center for Disease Control [19] found that the overall prevalence of depression in Puerto Rico was 16.8% in 2011, 16.3% in 2012, 18.8% in 2013, and 18.5% in 2014. More recent findings revealed that about 10.4% of the Puerto Rican adult population suffers from some form of mood disorder, while 12.5% of that population experiences some form of anxiety disorder [20]. On the other hand, comparison analyses between Puerto Ricans living on the island and Puerto Ricans living in the United States (USA) showed that Puerto Ricans from the island had similar rates of psychiatric disorders to Puerto Ricans living in the U.S. [21]. Puerto Ricans in the USA had specifically higher levels of anxiety and depression, but not psychiatric disorders in general, compared to those on the island. Furthermore, among young Puerto Ricans, 13.4% of adolescents were found to suffer from major depression, 8.3% from suicidal ideation, and 6.9% from behavioral disorders [22].

### 1.2. Instruments that Measure Depression and Anxiety in Puerto Rico

The most widely used measuring instrument in Puerto Rico to examine symptoms of depression in adults is the Beck Depression Inventory (BDI; BDI-II) [23]. This instrument was validated in Puerto Rico with 300 participants and obtained an internal Cronbach alpha consistency of 0.89 [9]. A revised version of the BDI was later developed in Puerto Rico according to the DSM-IV diagnostic criteria [24]. The psychometric properties of the instrument were examined with a sample of 351 Puerto Rican university students, and revealed an internal consistency of 0.88. Later, other Puerto Rican researchers assessed the reliability of the BDI-II and revealed an internal consistency of 0.91 [10]. In addition, the Rodríguez-Gómez Hispanic Depression Questionnaire was developed with a sample of Puerto Rican elders in 2003 [25]. In 2005, it was administered in children and adolescents, and obtained a Cronbach’s alpha for internal consistency of 0.82 [26]. That same year, the applicability of the Spanish version of the Zulf Self-Rating Depression Scale was studied in a sample of 258 Puerto Rican adults, and obtained an alpha coefficient of 0.85 [27].

The efforts made in Puerto Rico to developing and validating instruments to measure depressive symptomatology in the adolescent population have been remarkable. For example, in 2008 the Self-Efficacy Scale for Depression in Adolescents was developed and validated with 116 participants, and obtained a Cronbach’s alpha for internal consistency of 0.90 [28]. Ten years later, it was validated using a sample of 51 Puerto Rican teens with Type 1 Diabetes, and obtained a Cronbach’s alpha for internal consistency rate of 0.93 for the total scale and between 0.71 to 0.85 for sub-scales [29]. Other Puerto Rican authors validated the Inventory for the Spectrum Assessment of Depressive Symptomatology with 201 adolescents by revealing a Cronbach’s alpha for internal consistency of 0.98 [30]. Finally, preliminary data from the Children Depression Inventory-2 on 51 Puerto Rican teens revealed a reliability of 0.84 for the total scale [31].

Regarding instruments measuring anxiety symptomatology in Puerto Rico, the internal consistency of the Beck Anxiety Inventory (BAI) was evaluated in 2001, which obtained a Cronbach alpha of 0.94 [11]. Later in 2016, the psychometric properties of the BAI were evaluated, and revealed an internal consistency of 0.95 [10]. Finally, the Generalized Anxiety Scale-7(GAD-7) revealed a Cronbach’s alpha for internal consistency between 0.91 to 0.92 in studies with Hispanics in Puerto Rico [32,33].

### 1.3. Description of Depression, Anxiety, and Stress Scales

Lovibond and Lovibond [34], DASS-21 authors, intended to develop an instrument capable of measuring symptomatology associated with depression and anxiety, and that could simultaneously discriminate between these constructs. For the development of the instrument, the authors included clinical and diagnostic symptoms of depression and anxiety, and excluded symptoms that may be present in both disorders, such as changes in appetite. However, factorial analyses in their first validations yielded a third factor (stress) that, according to the authors, gathers symptoms associated with difficulty relaxing, nervous tension, irritability, and agitation [34]. In this way, the first version of the instrument, the DASS-42 (long version of 42 items) was born, which is one of the most widely used tools in the world to measure affective symptoms.

This instrument comprises three scales: (1) the depression scale, which measures hopelessness, low self-esteem, and low positive affection; (2) the anxiety scale, which evaluates autonomic arousal, musculoskeletal symptoms, situational anxiety, and the subjective experience of anxious arousal; and (3) the stress scale, which measures tension, agitation, and negative affection. The instrument items refer to the previous week and each item is classified into four Likert responses from 0, which means “nothing” to 3, which means “Most of the time”. A short version of the DASS was later developed, which has now been recognized as the DASS-21 [34]. This instrument contains seven selected items from each of the scales.

### 1.4. Psychometric Properties in Other Countries and Populations

DASS-21 has been translated into Spanish and validated into Hispanic populations, revealing adequate psychometric properties [12,13,14,15]. The short version has shown adequate psychometric properties in validation studies of adults, the general-population, and in clinical samples. Similarly, previous research indicates that DASS-21 has a strong internal consistency and provides an adequate distinction between anxiety and depression, relative to other existing measures [12]. The authors of the instrument reported that the scales had an adequate convergent and discriminatory validity [34].

Regarding the factorial structure of the instrument, the literature is broad and diverse, showing that factorial structures can fluctuate depending on the sociocultural context and the type of population in which the instrument is being administered. For example, in Latin American countries, DASS-21 has revealed to fit well with a three-factor structure [12,35,36]. This is consistent with findings found in 2,630 Asian participants from Malaysia, Indonesia, Singapore, Taiwan, and Thailand who, after removing three items, identified a three-factor structure (DASS-18) [37]. The study authors argue that the original DASS-21 may not be applicable in Asian samples and therefore suggest that the instrument could be presented differently. Also, in eastern regions such as China, Iran, and Greece, a three-factor model was best suited in a sample of non-clinical Greeks [38,39,40].

Moreover, in a study with American teenagers, a four-factor structure was found, involving a new factor called negative affection [41]. This is consistent with findings in a Portuguese community [42] and in a sample of Vietnamese adolescents [43], where they found an additional factor of psychological distress (negative affectivity). Also, a sample of patients with brain trauma found a four-factor structure, with distress overall being the fourth factor [44]. The authors of these studies concluded that psychological distress is a factor underlying depression, anxiety, and stress. Other studies with clinical and non-clinical samples in the USA [45], Brazil [46], and Italy [47] have found two-factor models in DASS-21. In fact, a recent systematic review underpins the plausibility of an overall factor underlying depression and anxiety [48]. Also, the authors support the potential usefulness of a two-factorial model. As documented, the factorial structure of DASS has evolved into a variety of structures depending on the context. Examples of this are DASS-14 [49] and DASS-9 [40].

It is notable that DASS-21 has not been used in experimental studies with Latinos in which the effectiveness of Cognitive Behavioral Therapy in anxiety disorders has been evaluated [50]. Nor was the use of DASS-21 identified in randomized studies conducted in Puerto Rico until 2015 [51]. However, the instrument was used in a pilot intervention study showing a Cronbach’s alpha for internal consistency of 0.94 for the total scale with 32 adults [18]. In relation to non-experimental studies conducted in Puerto Rico, DASS has revealed an internal consistency between 0.87 and 0.90 [16,17,52,53]. However, our literature review revealed that, so far, the factorial structure and construct validity of DASS-21 in Puerto Rico Hispanics have not been examined.

### 1.5. Purpose of the Study

The main purpose of this study was to examine the construct validity of the DASS-21 in order to determine whether it is able to adequately discriminate between symptoms of depression and anxiety in the Hispanic population in Puerto Rico. To achieve our goal, several construct validity analyses were performed using advanced statistics. Specifically, this study had three main objectives:

1. Analyze the factorial structure of the DASS-21 by using an exploratory factor analysis to identify the dimensions behind the 21 items.
2. Perform a confirmatory factor analysis to examine whether the original three-factor model has a good fit in Puerto Rico Hispanics and analyze whether the factors maintain adequate independence between them.
3. Analyze the convergent and divergent validity of the three DASS-21 scales using the extracted mean variance analysis.

## 2. Methods

### 2.1. Research Design

This study employed an instrumental design [54] by using exploratory and confirmatory factor analyses with structural equations to examine the construct validity of DASS-21. This study was approved by the Institutional Ethics for Research Committee of the Carlos Albizu University, San Juan Campus, Puerto Rico. The data compilation was carried out by using online questionnaires through the PsychData platform and posting a paid ad in the main social networks as a recruitment method: FB, Twitter, Google+, and WhatsApp, among other platforms. This ad redirected the participants to the online survey, where they read the informed consent, which notified them of the following: (a) the purpose of the study, (b) inclusion criteria, (c) the voluntary nature of the study, (d) possible risks and benefits, and (e) their right to withdraw from the study at any time. To guarantee the privacy and confidentiality of the participants, the questionnaires were completed anonymously, and they were able to print a copy of the informed consent.

### 2.2. Participants

The process for selecting participants was by non-probabilistic availability. The sample of this research consisted of 1,073 participants recruited electronically. In Table 1 we present the full sociodemographic distribution. The age of the participants ranged from 21 to 77 years of age with an average of 37.68 years and a standard deviation of 11.69.

### 2.3. Measurement

To identify the sociodemographic characteristics of the sample, we developed a general data questionnaire composed of relevant data such as age, sex, academic preparation, civil status, and annual income.

An abbreviated version of the Depression, Anxiety and Stress Scales (DASS-21) was used. This scale was developed by Lovibond and Lovibond [34]. The three-dimensional self-reporting scales assess the presence and intensity of affective states of depression, anxiety, and stress. Each item is answered according to the presence and intensity of each symptom in the last week on a 4-point Likert response scale, the limits of which are the answer nothing and the answer most of the time. Each scale has seven items and its total score is calculated with the sum of the items belonging to that scale and varies between 0 and 21 points. A higher score indicates a higher participant symptomatology. Items 1, 6, 8, 11, 12, 14, and 18 belong to the stress scale, items 3, 5, 10, 13, 16, 17, and 21 to the depression scale, and items 2, 4, 7, 9, 15, 19, and 20 to the anxiety scale.

### 2.4. Data Analysis

Once the data was collected, we analyzed it using the IBM SPSS version 24.0 statistical analysis system. Specifically, descriptive sample analysis, exploratory factor analysis, reliability analysis, and factor correlation analysis were performed. For exploratory factor analysis, the method of extraction of main axes with oblique rotation was used to identify the latent variables underlying the items. This adjustment procedure was used for two main reasons: (1) the main axis extraction method is not based on the normality scenario [55], and (2) oblique rotation is more accurate and provides more information than rotation octagonal [56]. For factor identification, we used two criteria: (a) each factor must explain 5% or more of the variance [57]; and (b) each item must have a factorial load greater than 0.30 in a single factor [58].

The STATA version 14.1 statistical program was used for confirmatory factor analysis, with the maximum likelihood estimation method and the corrections of Satorra and Bentler [59]. To evaluate the adjustment of the models, we used the following adjustment indexes: Chi-square test (χ^2^), root mean square error of approximation (RMSEA), Tucker–Lewis Index (TLI), Comparative Fit Index (CFI), and Akaike Information Criterion (AIC). RMSEA values less than 0.05 indicate an adequate adjustment of the model [60]. Likewise, CFI and TLI values greater than 0.90 represent an adequate adjustment of the model [60]. AIC was used to examine the parsimony and compare the models, where the model with the lower index shows a lower adjustment [61]. Meanwhile, the regression coefficients of each item on its respective factor should exceed 0.50 to be considered adequate [62]. The correlation between the instrument factors was calculated using Pearson’s product-moment coefficient (*r*). Values less than 0.35 were considered to be weak or low correlations, values between 0.36 and 0.67 were considered moderate correlations, values between 0.68 and 0.89 were seen as high correlations and, finally, values from 0.90 onwards were considered to be very high correlations [63].

In addition, following the recommendations of Fornell and Larcker [64], we examined the convergent and discriminatory validity of DASS-21 through the Average Variance Extracted (AVE). To support convergent validity, the AVE must be equal to or greater than 0.50, thus establishing that more than 50% of the construct’s variance is due to its indicators [65]. For its part, in order to determine the discriminatory validity of each dimension, the Maximum Shared Variance (MSV) and the Average Shared Variance (ASV) must be less than the value obtained from the individual AVE of each factor. Finally, confirmatory factor analyses were computed to compare the following five factor models of the DASS-21: (a) a one-factor model; (b) a one-factor model with depression and anxiety loading on the same factor; (c) a two-correlated-factor model with depression and stress items loading on the same factor; (d) a two-correlated-factor model with anxiety and stress loading on the same factor; (e) a three-correlated-factor model (original model).

## 3. Results

### 3.1. Structure Validity: Exploratory Factor Analysis

To determine the factorial structure of DASS-21 and identify the underlying dimensions behind its 21 items, several exploratory factorial analyses were performed. The first analysis showed a three-factor structure that explained 63% of the variance of the original data. The Kaiser-Meyer-Olkin (KMO) test supported the adequacy of sampling data for the analysis, KMO = 0.962. Bartlett’s sphericity test was significant, X^2^ (210) = 15,217.489, *p* < 0.001, indicating that the correlations between the reagents were significantly different from zero, thereby providing an additional indicator of the adequacy for factor analysis. However, when the distribution of items by factor was reviewed, we identified that most depression and stress items were grouped into the first factor, three depression items were accommodated in factor 2, and the seven anxiety items were grouped into factor 3 (see Table 2). In the item retention process, item 2 was removed by loading more than 0.30 into two factors. Items 10, 17, and 21 were also removed for negative charges on factor 2.

With the remaining 17 items, the second exploratory factor analysis was performed showing a two-factor structure that explained 59% of the variance of the original data, KMO = 0.961; χ^2^ (136) = 11,553.959, *p* < 0.001. Most of the depression and stress items loaded back into the first factor and six of the anxiety items loaded on the second factor. Item 8 was removed for loading in a non-corresponding factor. The remaining 16 items underwent a third exploratory factor analysis that again showed a two-factor structure that explained 61% of the variance of the original data, KMO = 0.958; χ^2^ (120) = 10569.397, *p* < 0.001. Finally, an exploratory factor analysis was performed where we eliminated all stress items with the intention of examining whether the anxiety and depression items discriminate against each other. The results showed a one-dimensional structure that explained 59% of the variance of the original data, KMO = 0.944; χ^2^ (136) = 6275.645, *p* < 0.001. This analysis provides evidence of the lack of discrimination between depression and anxiety items in Puerto Rico’s Hispanic community. None of the analyses replicated the factorial distribution of the original instrument version.

### 3.2. Structure Validity: Confirmatory Factor Analysis

We then performed a confirmatory factor analysis using the maximum likelihood estimation method and the Satorra and Bentler corrections [59] to verify whether the instrument maintains its theoretical and three-dimensional structure in Hispanics of Puerto Rico. The model examined was composed of three latent factors (anxiety, depression, and stress) with each item. The results showed an inadequate adjustment: χ^2^ = 2119.229 (186) *p* < 0.001, *RMSEA* = 0.10, *CFI* = 0.87, *TLI* = 0.86, χ^2^ sb = 1392.490 (186) *p* < 0.001, *RMSEA* sb = 0.08, *CFI* sb = 0.88, *TLI* sb = 0.86. These indices did not meet acceptable levels of adjustment [59]. In turn, we examined the regression coefficients of each item, which ranged from 0.51 to 0.83 (see Table 3).

### 3.3. Convergent and Discriminant Validity

Both discriminant and convergent validity were examined through the average variance extracted (AVE). This method indicates the variance explained by the construct in the items. The higher the value of the AVE, the lower the error variance. The AVE values obtained for the factors ranged between 0.52–0.56 (see Table 4). For the AVE to be considered as acceptable, the scores must be equal to or greater than 0.50 [64,65]. On the other hand, for there to be evidence of discriminatory validity, the MSV and the ASV must be less than the value obtained from the AVE. However, the results showed that the MSV and ASV values exceeded the AVE of all three scales (see Table 4). This means that the three scales share a substantial amount of variance with each other. This is confirmed by observing high correlations between latent variables (ranged between 0.86–0.88), as well as correlations of the direct scores (ranged between 0.77–0.80).

### 3.4. DASS-21 Alternative Models

**Figure 1:**
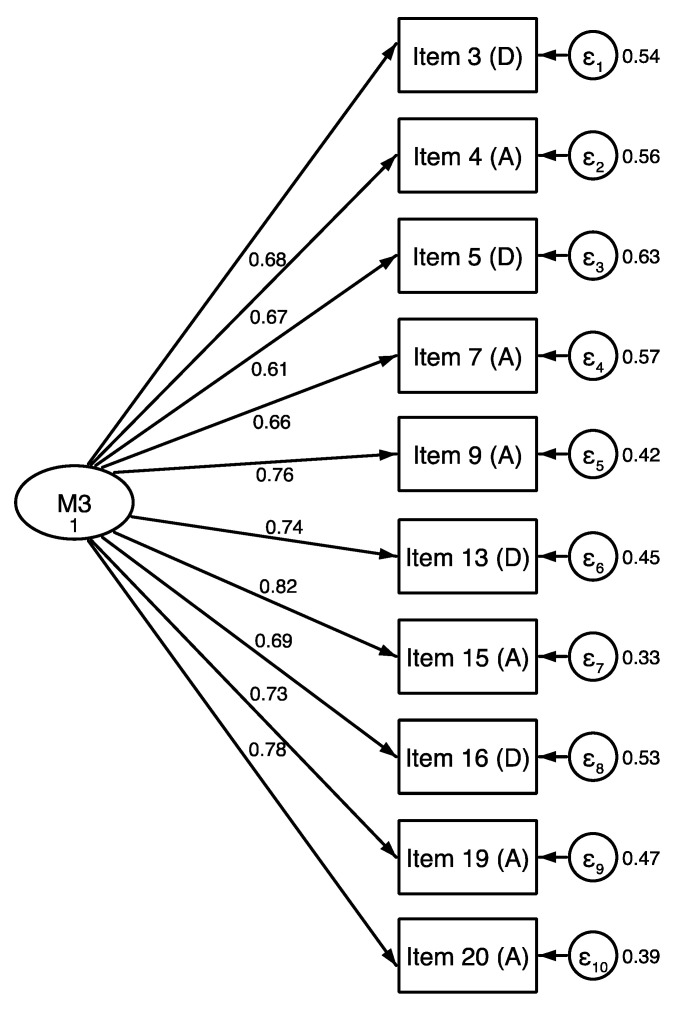
Model 3: One-factor model with depression and anxiety loading on the same factor.

Since the three-dimensional model of the DASS-21 did not obtain an adequate fit, other competitive model were examined. These alternative models were based on exploratory factorial analyses conducted in our study and previous research conducted in Spanish-American contexts [12,35,36]. Specifically, six competitive models were evaluated: the original model of three factors (M1); a unifactorial model where the 21 original items were loaded to one factor (M2); a one-factor model (10 items) with depression and anxiety loading on the same factor (M3; obtained from the 4th exploratory factor analysis of this study) (see Figure 1); a one-factor model (14 items) with depression and anxiety loading on the same factor (M4); a two-correlated-factor model with depression and stress items loading on the same factor (M5); and a two-correlated-factor model with anxiety and stress loading on the same factor (M6). The M1, M2, M4, M5, and M6 did not show an adequate adjustment to the data (see Table 5). The only model with adequate adjustment rates was the M3. The comparative analysis provides additional evidence on the lack of discrimination between depression and anxiety items in Puerto Rico’s Hispanic community.

## 4. Discussion

The high prevalence of mood and anxiety disorders in Latin America shows the need for a certain number of validated instruments in our cultural context, which allow the detection and treatment of these symptoms. However, the high comorbidity between depression and anxiety makes it difficult to develop instruments that properly discriminate between symptoms. For this reason, the main objective of this study was to examine the construct validity of the DASS-21 in order to determine whether it is able to adequately discriminate between symptoms of depression and anxiety in the Hispanic population in Puerto Rico. The results of this study demonstrated that the DASS-21 has serious psychometric deficiencies, especially in matters related to the construct validity, as well as convergent and discriminatory validity. These results will help us to offer a recommendation on its possible use in clinical and research scenarios with Hispanic populations.

However, our study has some limitations. First, the sample gathered was a convenience one, so it was not random. Second, it was not possible to establish the reliability of the instrument over time, as it could only be done through its components. However, the advanced techniques that were used in the study provided empirical strength to our results. Third, the procedure to collect the data was not standardized, and this may have affected the study means and increased the standard error. As for the strengths of the study, it is important to note that this study was the first in Puerto Rico to analyze the psychometric properties of DASS-21 in such a broad and heterogeneous sample. Finally, performing a confirmatory factor analysis with structural equations added value to our study. In a broader area, our study strengthens the importance of continuously and repeatedly reviewing the performance and psychometric properties of measurement instruments. In turn, it discredits the practice of some researchers in assuming that the psychometric characteristics of scales in social and behavioral sciences are consistent across time and culture. This implies the need to carefully and deeply analyze the psychometric properties of a measuring instrument in each population, culture, and/or country used.

Regarding our results, exploratory and confirmatory factorial analyses showed that DASS-21 does not replicate the three-dimensional structure or factorial distribution found in other research in international contexts [13,34,66,67]. This confirms that the internal structure of DASS-21 fluctuates depending on the socio-cultural context where the instrument is administered. Even the results of other studies with samples of Hispanics advocating for a three-dimensional model are questionable and inconclusive [12,35]. For example, in the relevant study of Chile, they carried out an exploratory factor analysis with a solution restricted to three factors, and found that five items presented significant or relevant loads in two factors and an item with a factorial weight that was below the expected level [12]. These items had to be removed in the validation process to perform further exploratory factor analyses only with the remaining items. For their part, in a study of Mexico, researchers performed an exploratory factorial analysis with varimax rotation that revealed six factors [35]. However, the authors decided to maintain the first three factors to preserve the theoretical consistency of the instrument. Of the 21 items, only 14 were grouped into three factors. The authors had to perform a new exploratory factorial analysis with these 14 items to confirm whether the three-dimensional structure was maintained. For these reasons, we suggest taking the conclusions of these two studies that preliminary state that the DASS-21 is reliable and valid for measuring anxiety and depression with caution. In contrast, our findings argue that the original version of DASS-21 may not be applicable in Hispanic samples and its three-dimensionality is highly questionable, so it cannot be said that the scales are three distinct measures in Hispanic populations.

On the other hand, we find high correlations between the three instrument scales, both in the correlation rates between the latent factors and between the direct scores of each scale. This finding is not surprising given that studies using clinical scales of anxiety and depression often reflect moderately high or high correlations between the two constructs [14,15,34,68]. This precisely indicates that the impossibility of adequately discriminating between symptoms associated with depression and anxiety is the biggest problem when using DASS-21 in Hispanic populations. This is confirmed by our analysis of discriminatory validity, which showed that the three scales share a substantial amount of variance with each other, subtracting evidence from the instrument’s construct validity. Also, despite the high comorbidity between symptoms of anxiety and depression in clinical populations, our findings should be interpreted as empirical evidence in favor of a one-dimensional structure in DASS-21. In fact, our analysis of competitive models showed that the only model that was properly adjusted was the one-dimensional model that collected all items from the anxiety and depression scales.

In theoretical terms, depression and anxiety manifest in a distinct way, but in empirical terms they are very difficult to distinguish by using of self-reports. Hence there is a need to develop valid and reliable clinical measurement instruments that facilitate the diagnosis and treatment of people who have simultaneous symptoms of anxiety and depression. In this case, it appears that the DASS-21 does not meet the requirements of such a need. So, if the one-dimensional structure was the most appropriate one to explain the data, what model can construct measures or examine this one-factor dimension? Some authors have suggested that depression and anxiety scales predominantly measure the common factor of negative affectivity [35,69]. Negative affectivity, referred to in some studies as psychological distress [42], reflects dispositional dimensions, where high negative affectivity is characterized by subjective affliction and displaced feelings, and low negative affectivity is characterized by the absence of these feelings [69]. In this sense, the use of the one-dimensional version of DASS could be justified by researchers or clinicians to identify the presence of negative affectivity in individuals. Notably, some studies in Puerto Rico have used it for this purpose [52,53]. However, it should not be used to discriminate or differentiate between symptoms of anxiety and depression in Hispanics, at least until there is greater psychometric evidence supporting this function.

In practical terms, the use of DASS-21 as an evaluation method in clinical scenarios is discouraged. The lack of construct validity demonstrated in this study highlights the difficulty of DASS-21 identifying symptomatology associated with different treatable mental disorders in psychotherapy. That is, the scores obtained in the original version of the instrument would not represent weight indicators that favor the psychological evaluation process. Regarding the internal consistency of the scales, our results reflected acceptable values of reliability, all of which were above what the literature suggested. However, the reliability of the DASS-21 should not be interpreted as evidence of construct validity. Reliability is about certainty and not truthfulness [58]. In this sense, the reliability rates of the three scales only allow us to know if the items measure the same phenomenon, which in this case could be some aspect of negative affectivity and not necessarily depression, stress, or anxiety.

We recommend administering the DASS-21 to another sample of participants to perform the cross-validation process again and test the factorial invariance of the instrument, as well as to evaluate the concurrent validity of the instrument using other scales that measure depression, anxiety, and stress. For example, BDI-II [70], BAI [71], or the Patient Health Questionnaire (PHQ-9) [72] could be used for the validity process. Likewise, we advise examining the properties of DASS-21 in a clinical population, as well as in an adolescent population in Puerto Rico. Finally, it would be interesting for future research to examine whether the Hispanic population of Puerto Rico has a particular psychosocial condition that explains the results of DASS-21 in Puerto Rico.

## 5. Conclusions

This study demonstrated that the DASS-21 has serious psychometric deficiencies, especially in matters related to construct validity, as well as convergent and discriminatory validity. The findings empirically demonstrated that the DASS-21 does not replicate the three-dimensional structure of the original instrument in Puerto Rico’s Hispanic community. The results also suggest that the internal structure of the instrument fluctuates depending on the socio-cultural context where the instrument is administered. In Hispanics, it seems that the instrument is better suited to a one-dimensional model that examines negative affectivity. Finally, this research confirmed the difficulty of the DASS-21 in properly identifying and discriminating between symptoms associated with depression and anxiety in Hispanic populations. It is recommended not to use DASS-21 with Hispanics in clinical and research contexts, at least until there is greater psychometric evidence.

## Figures and Tables

**Table 1:** Sociodemographic data of the sample.

	***n***	**%**
**Sex**		
Female Male	818 225	76.2% 23.8%
**Academic Preparation**		
High school or less Associate degree/technical Bachelor’s degree Master’s degree Doctoral degree	66 210 462 253 82	6.2% 19.6% 43.1% 23.6% 7.6%
**Civil Status**		
Marriage Single Cohabiting (free union)	566 187 320	52.7% 17.4% 29.8%
**Annual Income**		
$0–25,000 $26,000–50,000 $51,000–75,000 $76,000–100,000 $101,000 or more	588 320 108 36 21	54.8% 29.8% 10.1% 3.4% 2.0%

Note: *N =* 1073.

**Table 2:** Distribution of items in the four exploratory factorial analyses.

	1st Analysis			2nd Analysis		3rd Analysis		4th Analysis
	1	2	3	1	2	1	2	1
**Depression**								
Item 3	0.62			0.65		0.64		0.68
Item 5	0.41			0.42		0.41		0.61
Item 10		−0.79						
Item 13	0.66			0.72		0.70		0.75
Item 16	0.48			0.52		0.50		0.69
Item 17		−0.58						
Item 21		−0.85						
**Anxiety**								
Item 2	0.31		0.31					
Item 4			0.67		0.61		0.63	0.66
Item 7			0.82		0.84		0.79	0.65
Item 9			0.64		0.73		0.70	0.76
Item 15			0.58		0.70		0.71	0.82
Item 19			0.60		0.63		0.65	0.72
Item 20			0.53		0.72		0.73	0.78
**Stress**								
Item 1	0.76			0.76		0.77		
Item 6	0.62			0.64		0.64		
Item 8			0.67		0.67			
Item 11	0.56			0.60		0.59		
Item 12	0.86			0.89		0.90		
Item 14	0.68			0.70		0.69		
Item 18	0.79			0.82		0.80		

**Table 3:** Regression coefficients (*β*) on its respective scales, and confidence intervals.

Items	*β*	*95% C.I.*
**Depression**		
3. I could not seem to experience any positive feeling at all.	0.70	[0.67, 0.74]
5. I found it difficult to work up the initiative to do things.	0.60	[0.55, 0.64]
10. I felt that I had nothing to look forward to.	0.80	[0.76, 0.83]
13. I felt down-hearted and blue.	0.79	[0.76, 0.81]
16. I was unable to become enthusiastic about anything.	0.72	[0.68, 0.76]
17. I felt I was not worth much as a person.	0.80	[0.76, 0.83]
21. I felt that life was meaningless.	0.80	[0.76, 0.83]
**Anxiety**		
2. I was aware of dryness of my mouth.	0.51	[0.46, 0.57]
4. I experienced breathing difficulty.	0.67	[0.62, 0.72]
7. I experienced trembling (e.g., in the hands).	0.67	[0.61, 0.72]
9. I was worried about situations in which I might panic and make a fool of myself.	0.78	[0.74, 0.81]
15. I felt I was close to panic.	0.83	[0.80, 0.86]
19. I was aware of the action of my heart in the absence of physicalexertion.	0.74	[0.70, 0.77]
20. I felt scared without any good reason.	0.79	[0.75, 0.82]
**Stress**		
1. I found it hard to wind down.	0.64	[0.60, 0.68]
6. I tended to over-react to situations.	0.70	[0.66, 0.73]
8. I felt that I was using a lot of nervous energy.	0.72	[0.69, 0.76]
11. I found myself getting agitated.	0.82	[0.79, 0.85]
12. I found it difficult to relax.	0.80	[0.78, 0.83]
14. I was intolerant of anything that kept me from getting on with what I was doing.	0.75	[0.71, 0.78]
18. I felt that I was rather touchy.	0.80	[0.78, 0.83]

Note: *β* = standardized regression coefficients; *p* = significance; *95% C.I.* = 95% confidence intervals of regression coefficients.

**Table 4:** Means, standard deviations, alphas, omega coefficient, average variance extracted, and correlations.

	*M*	*SD*	*α*	*ω*	*AVE*	*MSV*	*ASV*	1	2	3
1. Depression	4.78	4.87	0.89	0.89	0.54	0.77	0.77	-	0.88 **	0.87 **
2. Anxiety	3.81	4.54	0.88	0.88	0.52	0.77	0.76	0.79 **	-	0.86 **
3. Stress	6.98	5.32	0.90	0.90	0.56	0.76	0.75	0.80 **	0.77 **	-

Note. *M* = Mean; *SD* = *standard deviation*; α = Cronbach’s alpha coefficient; *ω* = omega coefficient; *AVE* = average variance extracted; *MSV* = maximum shared variance; *ASV* = average shared variance; ** = significant correlations *p* < 0.001. The values on the diagonal represent the correlations between the latent factors, while the values below the diagonal represent the correlations of the direct scores.

**Table 5:** Goodness-of-fit test for analyzed models.

Model	χ^2^	χ^2^_sb_	*GL*	*RMSEA*	*RMSEA*_sb_	*CFI*	*CFI*_sb_	*TLI*	*TLI*_sb_	*AIC*
M1	2119.229	1392.49	186	0.10	0.08	0.87	0.88	0.86	0.86	46,023.094
M2	2748.703	1800.22	189	0.11	0.09	0.83	0.84	0.81	0.82	46,646.568
M3 *	791.755	482.499	76	0.09	0.07	0.92	0.93	0.91	0.91	30,085.567
M4	1323.558	796.913	77	0.12	0.09	0.86	0.84	0.87	0.85	30,615.370
M5	2442.991	1602.66	188	0.11	0.08	0.85	0.86	0.83	0.84	46,342.856
M6	2484.373	1631.30	188	0.11	0.09	0.85	0.86	0.83	0.84	46,384.237

Note. * = adequate adjustment; sb = Satorra–Bentler adjustments; χ^2^ = Chi-square test; χ^2^_sb_= Corrected Chi-square test; *GL* = degrees of freedom; *RMSEA* = root mean square error of approximation; *RMSEA_sb_* = corrected *RMSEA*; *CFI* = Comparative Fit Index; *CFI_sb_* = Corrected *CFI*; *TLI* = Tucker–Lewis Index; *TLI_sb_* = Corrected *TLI*; *AIC* = Akaike Information Criterion; All statistics χ^2^ and χ^2^_sb_ are significant, *p* < 0.001.
